# An Account of an Uncommon Case of Emphysema; and of an External Abscess, the Contents of Which Were Discharged by Coughing

**Published:** 1797

**Authors:** Edward Augustus Holyoke


XX. *An Account of an uncommon Case of Emphysema; and of an external Abscess, the Contents of which were discharged by coughing. By* Edward Auguſtus Holyoke, *M. D. F. A. A.* — From the *Memoirs of the American Academy of Arts and Sciences,* Vol. II. Part I. 4to. Boſton, 1793.

ON Tueſday, July 15, 1783, I was deſired by the gentleman who attended him, to viſit a boy of about twelve months old, who he told me had been moſt ſeverely handled by a peripneumony for ten days; but that a very uncommon tumour lately appearing upon the child, made him deſirous I ſhould ſee him.

Upon viewing the patient, I found a large, ſoft, elaſtic, flatulent tumour, evidently crackling under the fingers when preſſed upon, as tumours ariſing from air in the cellular membrane uſually do. This tumour ſhewed itſelf all at once on the preceding evening, about ſeven or eight o'clock, as the child lay in its mother's lap, during a violent fit of coughing,

 on on one ſide of the neck, near the right maſtoid muſcle; and by ten o'clock the next morning, when I firſt ſaw it, it had ſpread acroſs the neck, and up by the left ear, under the ſcalp, ſo as to cover the whole extent of the crotaphite muſcle on that ſide; on the left ſide it extended no farther up than the ear; downwards, it ſpread on the breaſt below the paps on both ſides, and to each axilla, and was evidently increaſing, eſpecially upon coughing.

The pulſe was frequent, the fleſh hot, the reſpiration deep, frequent, and laborious to a great degree; the cough frequent and violent; and the mouth lined with aphthæ; but none of theſe ſymptoms were increaſed (as I was informed) ſince the Emphyſema had taken place: the child appeared in the utmoſt danger.

About one o'clock the ſame day, I ſaw the child again; the ſymptoms continued much the ſame; but the flatus had ſpread on the left ſide of the head up to the vertex; and farther down upon the breaſt; and farther round than the axillæ.

The next morning, Wedneſday, the 16th, the Emphyſema occupied a larger ſpace upon the head, though chiefly on the left ſide ſtill; had extended over both ſcapulæ; and had got farther down on the breaſt, and indeed covered the whole thorax on the fore part, and on the left ſide paſſed over the oblique abdominal muſcles down to the groin. The child had now a more cadaverous look; the hands were purpliſh, and the pulſe plainly lower and more ſunk; the difficulty of breathing ſtill kept up; and we expected he would ſoon expire.

Thurſday, 17th, A. M. Matters in much the ſame ſituation as yeſterday, only the tumour now extended over both ſides of the abdomen, but did not paſs over the recti muſcles; covered almoſt all the back, and on the head had paſſed over the vertex, and now covered the whole right ſide.

Friday, 18th, the child ſtill alive; the dyſpnœa, cough, &c. ſtill continued. He had taken no medicine for twenty-four hours paſt, nor had ſwallowed any thing but a little drink. The tumour now covered the whole trunk, except a ſmall area round the navel, and a narrow ſtripe both above and below it, upon the linea alba, which were free from all ſwelling; the whole neck was puffed up, and the head under the hairy ſcalp every where, except the back part, where the air ſeems to have been prevented from inſinuating itſelf, by the preſſure of of the head, as it lay upon the pillow; the face was every where free, as alfo both the upper and lower limbs. This evening the child died; but, to our great mortification, no perfuafions could prevail upon the mother to permit the body to be infpected. The appearance of the tumour upon the dead body was much the fame the next morning as it had been before death.

To account for thofe uncommon appearances, I think we muft fuppofe a communication fomewhere between the cavity of the lungs and the cellular membrane; and as the firft appearance of Emphyfema took place in the neck, upon a violent fit of coughing, it feems highly probable that this communication was formed by a rupture of the membranes of the afpera arteria, fomewhere between its cartilages*; and thus gave paffage to the air from the lungs into the adjacent cellular membrane, at every expiration; and as the cough was very violent, the air would, at every fuch effort, more efpecially, be forcibly impelled through this opening, and

* Poffibly a fmall abfcefs might be formed between thefe membranes, and fo, by weakening them, occafion their burfting, upon a violent exertion in coughing.

thus extend itſelf wherever this membrane extended; at firſt indeed more rapidly, but ſtill continue to extend, till the reſiſtance which the air met with, in paſſing out at the opening, was equal to the force by which it was expelled from the trachea in expiration or in coughing.

This ſolution of theſe appearances cannot be aſcertained, as we were not allowed to open the body, and whether it will be thought admiſſible, I cannot determine; but as another caſe which fell in my way not a great while ago, may throw ſome light upon this, I will take the liberty to relate it.

A man, about fifty-three or fifty-four years old, of a thin habit of body, labouring under a very bad cough, attended with a hectic fever, profuſe ſweats, &c. had a large tumour formed upon the upper part of the thorax on the left ſide, extending from the ſhoulder, all along the lower edge of the clavicle, to the ſternum, about the breadth of a man's hand. This tumour had all the appearance of a large abſceſs; it was accordingly treated as ſuch, and ſuppuration ſeemed to be coming on as uſual; but on removing the dreſſings one day, I found the tumour (though the ſkin remained whole) leſs prominent to the eye, flabby to the touch, and the pain and inflammation abated. I was now at a loſs what to make of the caſe, as the abſceſs ſeemed too far advanced to expect diſcuſſion. While I was thinking of the matter, the patient aſked me, " What could occaſion " that blubbering noiſe (as he expreſſed him- " ſelf) in the ſore? Upon which, applying my ear near the part where he perceived the noiſe, I plainly heard a whizzing, and, as he termed it, a blubbering noiſe at every breath, exactly reſembling ſuch as ariſes from the ruſhing of air through a ſmall orifice. This orifice appeared to be juſt under the left clavicle, but nearer to the ſhoulder than the ſternum. Upon viewing the part attentively, a ſmall dilatation and contraction were perceptible upon expiration and inſpiration; and the part was evidently puffy and flatulent to the touch. At this time the cough was urgent, and the expectoration very copious.

From this time the tumour, inflammation, and hardneſs ſubſided; the noiſe in breathing gradually leſſened, till it ceaſed; and by the aſſiſtance of pectoral medicines, the bark, &c. the hectic and cough after a while left him, and with them the ſweats, &c.; his appetite returned, returned, and he recovered his ſtrength, though ſlowly, and is at this time in tolerable health.

In this caſe I think it certain that the inflammation penetrated to the lungs, which, no doubt, adhered to the pleura in this part; and the abſceſs burſting inwardly, the matter was diſcharged through the trachea by the aſſiſtance of the cough, which was at this time very conſtant; but the cavity of the lungs having now a communication with the cavity of the abſceſs, ſome of the air from the lungs would paſs at every expiration into this cavity; but would not diffuſe itſelf in the cellular membrane, and produce general emphyſema in this caſe, as in the caſe firſt mentioned, probably becauſe the inflammation of the cellular membrane, which ſurrounds all abſceſſes, and limits their extent, muſt have formed a barrier impenetrable by the air, as it ruſhed out of the lungs into this cavity; and of courſe the whole of what was thrown into the cavity of the abſceſs at each expiration, would be drawn back again into the lungs at the next inſpiration, and thus the ſurrounding parts might eſcape tumefaction; and this paſſing and repaſſing of the air will fully account for the noiſe which the patient complained of.

